# Neural Dynamics of Attentional Cross-Modality Control

**DOI:** 10.1371/journal.pone.0064406

**Published:** 2013-05-16

**Authors:** Mikhail Rabinovich, Irma Tristan, Pablo Varona

**Affiliations:** 1 BioCircuits Institute, University of California San Diego, La Jolla, California, United States of America; 2 Grupo de Neurocomputación Biológica, Dpto. de Ingeniería Informática, Escuela Politécnica Superior, Universidad Autónoma de Madrid, Madrid, Spain; Georgia State University, United States of America

## Abstract

Attentional networks that integrate many cortical and subcortical elements dynamically control mental processes to focus on specific events and make a decision. The resources of attentional processing are finite. Nevertheless, we often face situations in which it is necessary to simultaneously process several modalities, for example, to switch attention between players in a soccer field. Here we use a global brain mode description to build a model of attentional control dynamics. This model is based on sequential information processing stability conditions that are realized through nonsymmetric inhibition in cortical circuits. In particular, we analyze the dynamics of attentional switching and focus in the case of parallel processing of three interacting mental modalities. Using an excitatory-inhibitory network, we investigate how the bifurcations between different attentional control strategies depend on the stimuli and analyze the relationship between the time of attention focus and the strength of the stimuli. We discuss the interplay between attention and decision-making: in this context, a decision-making process is a controllable bifurcation of the attention strategy. We also suggest the dynamical evaluation of attentional resources in neural sequence processing.

## Introduction

Attention, as many cognitive functions, arises from integrated processes in distributed networks of interconnected brain areas [1]. From this perspective, attention can be viewed as a higher-order process that emerges from the interactions of complex dynamical modes (structures) that are functionally united by a common cognitive task. This process - depending on the goal or the stimuli - focuses limited resources on one or a few tasks. Attention is a core property of all perceptual and cognitive operations. Given the limited capacity to process competing options, attentional processes select, modulate, and sustain the focus on the information most relevant to perform a cognitive task or drive behavior. External attention refers to the selection and modulation of sensory information, e.g., selecting locations in space, instants in time, or modality-specific inputs. Internal attention refers to the selection, modulation, and maintenance of internally generated information (e.g., task rules, responses, long-term memory, or working memory). Working memory, in particular, lies closest to the intersection between external and internal attention (see for review [2]). Attention and working memory cannot operate without each other. First, working memory has a limited capacity [3], [4], and thus attention determines what will be encoded and processed. Second, memory from past experience guides what should be attended. Brain areas that are important for memory, such as the hippocampus and medial temporal lobe structures, are recruited in attention tasks, and memory directly affects frontal-parietal networks involved in attentional control (e.g. see [5]). Working memory and attention are thus intimately related such that working memory encoding and maintenance reflects actively sustained attention to a limited number of mental modalities (see also [2]).

Experimental neuro- and cognitive- science is often based on the implicit premise that the brain mechanisms underlying perception, emotion and cognition are well approximated by steady-state measurements of neural activity. Recently a new paradigm has been unfolded in the study of brain dynamics departing from stable transient activity in neural networks [6]–[10]. Transients have two main features: (1) they are resistant to noise and reliable even in the face of small variations in initial conditions, (2) transients are input-specific, and thus convey information about what caused them in the first place. This new dynamical view manifests a rigorous explanation of how perception, cognition, emotion and other mental processes evolve as a sequence of metastable states in the brain, suggesting new approaches to the diagnostics of mental diseases and revealing the origin of many phenomena observed experimentally. One of such widely discussed phenomenon is the limitation of information processing resources in the brain. Here we will focus on two well-known of examples: limited attentional resources and low working memory capacity [11].

Neuroanatomical, physiological and modeling efforts suggest that attentional control is mediated by a variety of local-circuit inhibitory neurons, distributed throughout all layers and areas of the cortex (e.g. [12], [13]). The model of attentional control that we present in this paper relies on inhibition and focuses on sequential cognitive or behavioral action. We make several assumptions that simplify the complex attention control problem in order to appreciate how inhibitory mechanisms of attention dynamics give rise to temporary changes in cognitive multimodal information processing. In particular, we will focus on attentional control of sequential cognitive tasks that integrate different modalities. In fact, we provide here a mathematical formulation of the seminal ‘biased competition attention theory’ [14]–[16].

Because attention is a dynamical process, there is a need to build models that take into account dynamical features and bifurcations in order to make predictions based on the theoretical analysis of experimental observations. In our view, this type of modeling must rely on concepts such as the stability of sequential transient dynamics. Within this perspective, we can address the origin and critical role of the limitation of cognitive resources.

To understand the origin of the limited resources of information processing in the brain, it is necessary to go from the subjects to the processes, from kinematics to dynamics. For example, it is important to see that the capacity of working memory is not just a number, but a specific characteristic of a dynamical process – the working memory recall. If this process is unstable, the processing of mental information is ruined. To describe the stability of mental information processing that is related to working memory, it is necessary to use specific dynamical images and analyze their characteristics. It is important to emphasize that multimodality cognitive coordination or multisensory coordination – i.e. the binding of sequences – is one of the most powerful strategies to solve complex cognitive tasks or to build complex behavior. In a general case, both bottom-up and top-down processes are important for understanding the attention-sequence binding interaction: (i) the modulation of the attentional pre-selection of subsequent goals by a sequential performance of cognitive tasks [17], and (ii) attentional control of the binding of different modalities with a sequential temporal structure.

In what follows, we build a simple but general enough dynamical model to describe attentional control of sequential multimodal performance of overlapping and concurrent tasks. With different parameters one can build specific models for the description and analyses of the dynamics of diverse cognitive processes. We concentrate here on the investigation of the attentional control of sequential cognitive tasks that integrate different modalities.

## Materials and Methods

### Attentional state space

**Figure 1:**
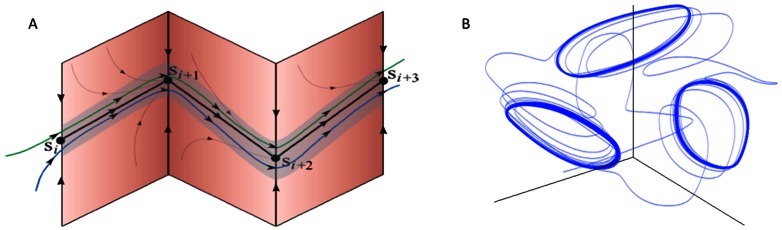
Chain of metastable states representing cognitive informational items in the state space. A: sequence of static metastable states –the mathematical image of these items is a saddle fixed point. One can see the trajectories in the neighborhood of the chain, which illustrates its stability [22], *S_k_* denotes the *k-*th informational item. B: sequences of dynamical metastable states – the mathematical image of these informational items is a saddle limit cycle. The chain of items can be open as in Fig. 1A, or closed as in Fig. 1B.

Let us introduce the state space as a space formed by a finite number of variables as functions of time. Such variables characterize different cognitive modalities. Generally, the cognitive information processing in these variables is represented by trajectories and includes different kinds of dynamical objects such as fixed points, limit cycles and stable manifolds in general (for details see [18]). Usually information processes in the brain are transient and can be considered as a temporal sequence of intermediate states possibly with their own fast intrinsic dynamics. In many cases such states represent individual informational items with finite lifetime. Here we will use the mathematical image shown in Fig. 1 to represent such transient information processes involved in attentional tasks. This image is a chain of meta-stable states represented by saddle fixed points or saddle limit cycles connected to their neighbors by unstable trajectories called separatrices. Such chain is stable if the contraction of the state space volume around the chain is stronger than the stretching along the unstable separatrices [18]–[22]. In this case, the trajectories that once enter the vicinity of the chain become prisoners and cannot leave this volume which is named as Stable Heteroclinic Channel – SHC (see Fig. 1A, [7], [20]).

### Finite resources

Let us consider the case when attention is guided by working memory (WM) [23]. Working memory capacity is finite. To understand the origin of informational resource limitations in the brain, it is necessary to analyze the conditions for the stability of the informational chain – the sequence of the informational items – and to estimate a realistic number of items that can be used for the cognitive processing. Such kind of estimation was done by Bick and Rabinovich for the analyses of working memory capacity [4]. In this study the authors considered a WM excitatory-inhibitory network that is able to dynamically sustain a finite number of information items. Their main result can be summarized as follows: for a fixed excitation level, the stability condition means that the level of inhibition increases exponentially with the number of items that can be recalled from WM without order mistakes. This value is not too high and is traditionally estimated in 7±2 items. This is the core of the information processing stability concept that helps to reveal the origin of the limitation of information processing resources in the networks responsible for attention and WM processes that are strongly interconnected [24], and even can provide an estimation of their capacity.

### Attentional network and global modes

Focusing attention requires the dynamical activity of inhibitory networks in the brain, which helps blocking incoming stimuli that are unrelated to a specific cognitive task or behavior. Several parts of the brain form together such networks - primarily those located in the frontal lobe and the parietal lobe of the brain [25]–[27]. More specifically, the mechanism of directed attention involves the prefrontal cortex (PFC), the anterior cingulate cortex (ACC) and the brain stem’s basal ganglia. The function of the PFC can be understood in terms of representing and actively maintaining abstract information such as goals, which produces two types of inhibitory effects on other brain regions. Inhibition of some subcortical regions has a directed global form, with prefrontal regions providing contextual information relevant to when to inhibit processing in a given region. Inhibition within neocortical (and some subcortical) regions has an indirect competitive form, with prefrontal regions providing excitation of goal-relevant options [28]. Authors in [29] also suggest that the right inferior frontal cortex (IFC) plays a specialized role in response inhibition. It seems that this region plays a key part in the integration of bottom-up response-related information and facilitates goal-directed behavior [30].

**Figure 2:**
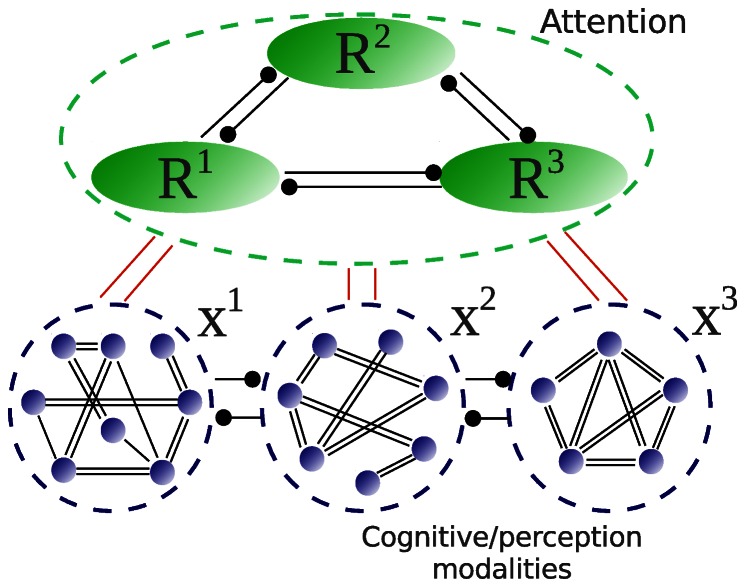
Architecture of the attention mode interaction in the case of three modality processing (*X^1^, X^2^, X^3^*). *R^1^, R^2^, R^3^* represent attention resource modes corresponding to these modalities. Black circles mean inhibitory connections.

We are going to model the dynamics of attentional inhibitory networks together with sequential multimodal mental activities in the framework of a global mode interaction approach [7], [20]–[22]. The model described below, invariant to different temporal scales, is based on accepted principles in attentional dynamics, mainly “the capacity of information processing is limited”. To investigate the dynamical mechanisms behind the cooperative activities of different modalities under attentional control, our general model of focused attention is implemented based on both bottom-up and top-down information flows (see Fig. 2 and also [31]).

### The Model

To build the model we will rely on three ideas that have been suggested by brain imaging experiments, multi-electrode recordings and computer experiments. First, to separate the spatial structures that correspond to cooperative ensembles of distributed neuronal clusters or spatial modes with a time dependent excitation level in each mode. Second, to focus on low-dimensional dynamics – i.e., to analyze the dynamics of a reasonable number of modes or first principal (independent) components of neural activity (see, for example, [32]–[37]). Finally, we will build an effective model as simple as possible.

To implement these ideas, let us represent the spatio-temporal dynamics of the resource field 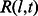, specifically attention, as the superposition of several attentional modes 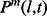: 
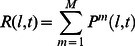
(1)



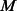
 is the number of interacting modes that compete for attentional resources. Under mode we understand the metastable composition of elements from different brain areas that are inter-correlated for the performance of a specific cognitive task. One of the typical mechanisms of such correlation is transient neural synchronization. We wish to describe the competitive dynamics of attentional modes using a canonical ecological model – Lotka-Volterra (LV) type equations with minimal, e.g. square nonlinearities:
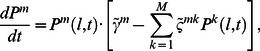
(2)


Where 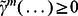 are step-like functions that represent the switching-on/switching-off of the *m*-th mode by excitatory input and 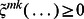 characterizes the inhibitory connections between attentional modes. They both depend on external and internal parameters (see below).

The LV model is pretty universal for the description of the dynamics of nonequilibrium dissipative systems and many other models can be written in such LV form after some recasting (see, for example [38]). Even equations with higher nonlinearities (cubic, etc.) after introducing new variables can often be written in the LV canonical form. The LV model demonstrates in a wide area of the parameter space a stable transient behavior whose mathematical image is a stable heteroclinic channel (SHC), and in fact it is a normal form for the analyses of local bifurcations of SHCs [39].

Now let us use the second idea: the separation of the space/time coordinates:
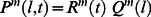
(3)


Here 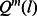 is a discrete spatial structure where 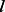 represents the set of discrete spatial elements from brain groups involved in these modes which behave coherently in time. The characteristic time scale of such coherentization or synchronization is 100-200 ms (see, for example [40]). This means that the dynamics of the metastable states –modes– has to be slower.

After the substitution of (3) in equation (2) and the summation on the spatial coordinate *l* we can get a model for 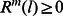 that describes the cross-modality attention dynamics:
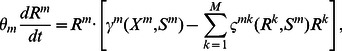
(4)


Here 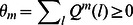, characterizes the time scale of the *m*-th attentional mode, 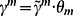 is the level of excitation of the *m-*th attentional mode by sensory or internal stimuli and the cognitive task, 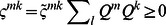. Because the modes 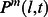 in a first approximation are independent, 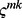 have a small value that corresponds to our approach - 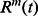 is a slow function of time compared with the coherentization process among the neuronal elements that form a mode.

The equations for the description of the dynamics of the cognitive/sensory multimodality fields can be obtained in the same way:
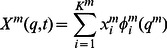
(5)


Here 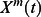 represents the spatio-temporal dynamics of the *m*-th cognitive modality field, where 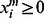 is the *i*-th mode of this field. The function 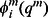
characterizes the spatial structure of the *i-*th mode associated with the *m-*th cognitive/perception modality, 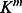 is the number of interacting modes inside the *m*-th cognitive modality, and 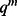 is an index for the neuronal groups that form the modes in the *m*-th modality.

Finally, the general dynamical model for 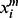 can be written in the form:
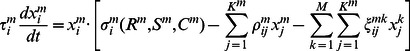
(6)


Here 
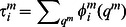
, and 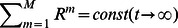, 
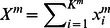
, 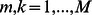; 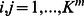; *S* is the vector of sensory inputs and *C* is the vector of cognitive inputs, 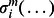 in the general case are step-like functions that represent the switching-on/switching-off of the *m*-th mode by excitatory input, 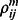 and 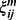 are connection matrices between modes of the same modality and between different modalities, respectively, 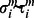, and 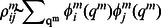, 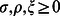.

Functions 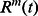 and 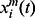 are the variables that form the informational state space of the dynamical model that we are looking for. The activation of different attentional modes means the activation of different sets of neuronal groups in the attentional network. For example, authors in [41] have shown based on the analyses of fMRI data that different attentional strategies correspond to the activation of different parts of the human parietal cortex.

This canonic excitatory-inhibitory model (4), (6) of an attentional network (in the case of nonsymmetric inhibitory connections) satisfies the information processing stability principle. It is possible to prove in the general case for *m*>2 that after a short transient period all system’s activity happens in the vicinity of the unstable separatrices of the metastable states (Afraimovich, 2012, private communication). Below we will show it for the case *m* = 3. This is a general enough case for a realistic number of modalities that share attention.

Feldman and Friston recently suggested that attention dynamics might be understood as inferring the level of uncertainty or precision during hierarchical perception. They illustrated this idea using neuronal simulations of directed spatial attention and biased competition [42]. Such approach is also related to moving from a multi-dimensional state space to low-dimensional manifolds.

## Results

### Stimulus dependent attentional control strategies: Low-dimensional dynamics and model parameters

For the understanding of top-down regimes of attentional control we have to answer the question: what kind of intrinsic attention dynamics is structurally stable, i.e., does not change qualitatively with a small variation of the control parameters of the attentional network?

**Figure 3:**
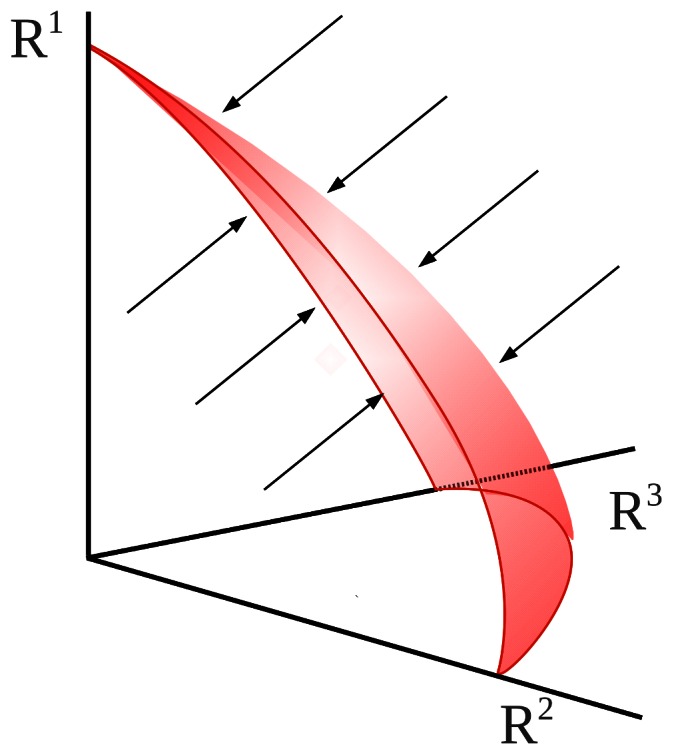
"Side" view of the simplex of a three-dimensional competitive system (4) (for the mathematical definition see [43]).

It is possible to see from the analyses of model (4) that important attentional events occur just in a restricted area of the state space. Figure 3 illustrates this for three interacting modalities. One can see that when time increases all trajectories are attracted by a quasi-two-dimensional volume. This volume is the vicinity of a two dimensional surface that is named *simplex.* On the other hand, the simplex itself has a finite size limited by boundary separatrices (see Fig. 3).

Thus, all robust attention activity is described by the trajectories, which after a short transient are disposed in a low dimensional finite volume. This finite volume in the informational state space is the mathematical representation of the limitation of information processing resources.

Let us now use the knowledge about intrinsic attention dynamics to answer the following question: how does competitive attention influence multitask/multimodality cognitive performance? Current literature suggests that, both multimodality interaction or integration and attentional control take place and can act on many levels of the brain structural hierarchy (for reviews see [44], [45]). Because multimodal objects and events activate many sensory cortical areas simultaneously, it is possible that reciprocally modulated activities of different modalities take place even at the level of primary cortical areas. Several experiments indicate that such modulation occurs because of mutual inhibition. In particular, Iurilli et al. showed that the auditory cortex activation by salient stimuli degrades potentially distracting sensory processing in the visual cortex by recruiting local, translaminar, inhibitory circuits [46]. Although the precise mechanisms of cognitive control of multimodal activities are not completely known, it is possible to say that they operate in a top-down manner through attention mechanisms [47], [48]. Based on the existing understanding of the interplay between attention and multimodal interaction, we can use a general dynamical model (4), (6) of these processes. We discussed above the logic of this model (see Fig. 2).

To analyze the effects of top-down attentional control based on the model (4), (6) we have to first specify the functions 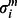 as
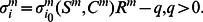
(7)


Here we will show results using network models composed of six modes. The parameters 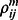 used to model the 6-mode networks that represent each modality are 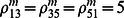; 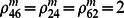; 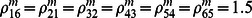; 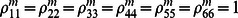; allowing ±2% variability in these values for each of the networks. The values of 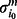 were chosen in these specific simulations as 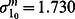, 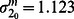, 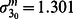, 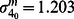, 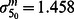, 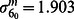. In this example, the binding connections between the three modalities were built with 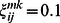 only between odd-numbered corresponding modes. Initial conditions for 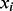 were randomly set for each modality (see [49]). The parameters 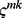 used to model the attention dynamics between modalities are 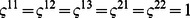, 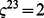, 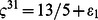, 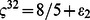 and 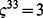; with 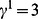, 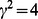, 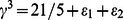 (see also [50]).

### Typical attention strategy

**Figure 4:**
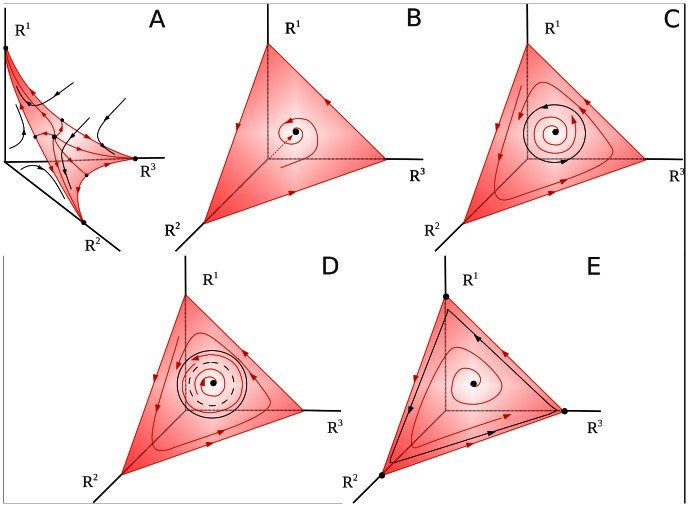
Examples of different attention crossmodality dynamics for Eq. (4) in the case of *M* = 3. Panel A corresponds to the performance of cognitive tasks that need complete attention focused on one of the tasks – one can see the multistability on the simplex (three stable fixed points whose basins of attraction are bounded by separatrices of the saddle fixed points). Panel B corresponds to the coexistence of cognitive tasks that need just a little attention each – the image is a stable fixed point that is a global attractor on the two-dimensional stable manifold (simplex, c.f. Fig. 3). Panel C shows that a stable limit cycle emerges on the simplex, this is the mathematical image of periodic changes of attention levels focused on different modalities. Panel D represents dynamical attention bistability – the coexistence of two attractors, i.e. stable fixed points and stable limit cycle. Finally, panel E shows a stable limit cycle in the vicinity of a closed heteroclinic contour which represents the sequential switching of attention among three different modalities. For mathematical details of these bifurcations see [51].

The possible attentional control strategies are formed during the human development and learning stages. The selection of the strategy depends on the environmental conditions and the cognitive task. In our model (4) this is formally expressed by the value of control parameters 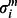, and 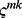. In particular, in the case of strongly competitive and equivalently important tasks these values are 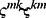 and 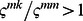. Depending on the initial conditions, whole attention can be focused on only one of the modalities. This type of dynamics is named as multistability (see Fig. 4A).

When competitive modalities are not equally competing with each other (e.g. non-reciprocal competition) another kind of multistability appears. The dynamics of *R^1^, R^2^, R^3^* is much richer. In particular, the stable fixed point (focus in Fig. 4B) becomes unstable and, as a result of a Hopf bifurcation, a stable limit cycle appears (Fig. 4C). It is also possible that the stable fixed point and the stable limit cycle coexist (see Fig. 4D). In this case a short external stimulus is able to change the strategy of the attentional control – instead of a static distribution of attentional resources (stable fixed point in Fig. 4D), the system performs a rhythmic modulation of the cognitive activities (stable limit cycle in Fig. 4D).

### Stimulus dependent timing and attention switching

**Figure 5:**
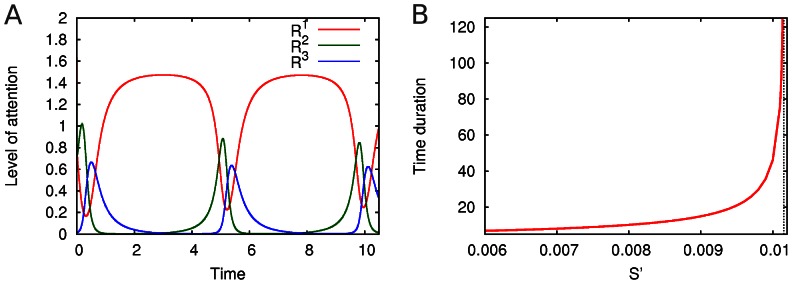
Competition between three attention modes. In panel A, we observe that the main focus of attention is in the first mode. The parameters 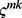 used to model the attention dynamics between modalities are 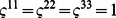, 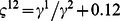, 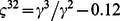, 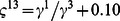, 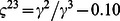, 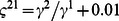, 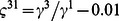; and 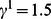, 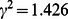, 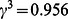. In panel B, we see that the time during which the system is able to keep attention on a specific modality depends on the strength of the stimuli. The figure shows the time duration of the regular switching between attention modes for the same system described in panel A, except for 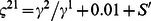 and 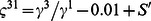 with 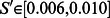.

The dynamical features of sequential attention switching between different modalities depend on the external or internal stimuli. If, for example, the environment changes so that modality 1 needs more attention, then the inhibitory suppression of modalities 2 and 3 by the modality 1 becomes stronger - 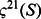 and 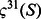 become larger and thus the attention strongly focuses on modality 1, and correspondingly the duration of this attention increases as shown in Fig. 5A. Let us emphasize that the time during which the system is focused on a given modality (e.g. modality 1) explosively increases with the strength of the stimuli corresponding to this modality (see Fig. 5B).

This result is easy to interpret on the following example. It is well known from daily life and supported by experimental and clinical evidence that pain demands attention and thus influences the performance of cognitive tasks including the attraction of attention for longer and longer time. This happens because pain changes the activity of many cortical areas that are involved in cognitive activities [52], [53]. Suppose we are driving a car and have some pain in the back. If the pain is weak, we can control the road and even listen to the radio. When pain increases, attention focuses on the pain for longer and longer time intervals and, when it is higher than some critical value, we are automatically completely concentrated on our body and are forced to stop the car. This is the well-known phenomenon of “interruptive function of pain” [54].

**Figure 6:**
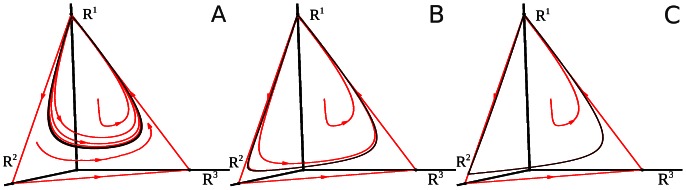
Phase portraits of system (4) corresponding to the time series represented in Fig. 5A for the following parameters: 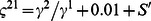 and 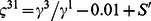 with 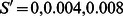, for A, B and C, respectively.

**Figure 7:**
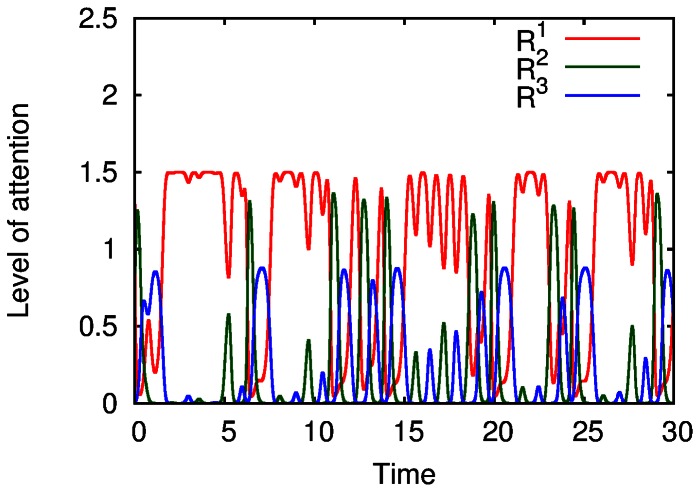
Irregular attention switching under the action of a periodic stimulus with main focus on the first mode. The parameters 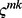 used to model the attention dynamics between modalities are 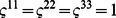, 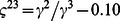, 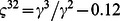, 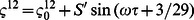, 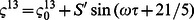, 
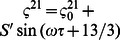
, 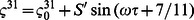, 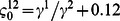, 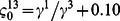, 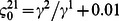, 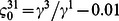, 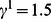, 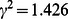, 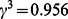, 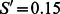 and 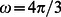.

The evolution of the limit cycle that represents the sequential attention switching when increasing the parameter *S’* can be seen in Fig. 6. It is interesting that under rhythmically changing stimuli the attention switching between different modalities can become irregular (in mathematical language chaotic), see Fig. 7, which can be related to irregular perceptual alternations [55].

### Sequential switching of attention modifies binding

**Figure 8:**
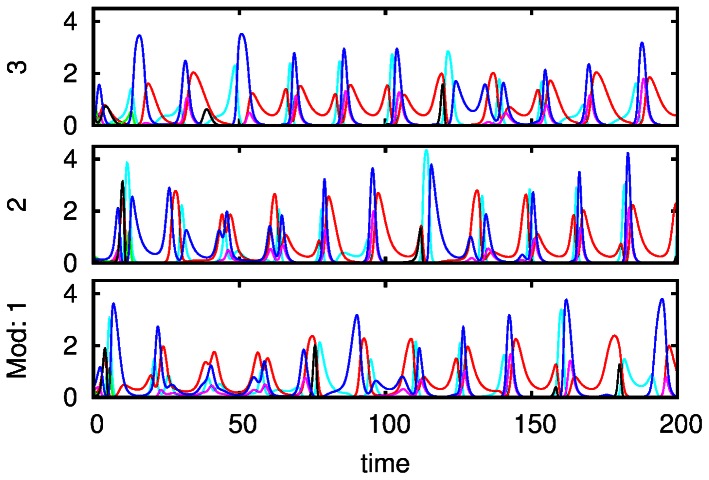
Time series of three modality attention dynamics. Three modalities under attentional control (each of them contains six modes). The dynamic of attention is represented by a limit cycle on the simplex in Fig. 4 C–E with 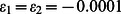 (the other parameters are set as indicated in Fig. 7).

The simulations of our model show that attentional dynamics are important for the performance of the binding process. In such performance maximum attention is sequentially focused on different modalities (see the time series in Fig. 8). Rabinovich et al. have shown that multiple sensory modalities can form a new dynamical object in the space of multimodality fields – this object is called a network of heteroclinic channels [18], [22], [49]. The emergence of this dynamical object depends upon a moderate degree of inhibitory interaction between the fields – a process that we will associate with inhibitory binding.

**Figure 9:**
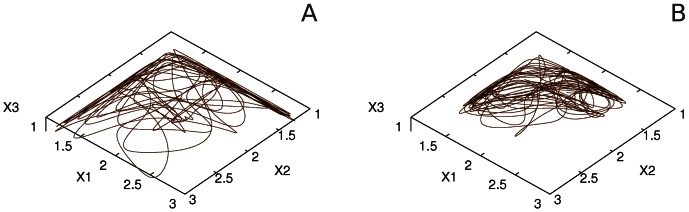
Phase portraits of three modalities dynamics in the *X^1^, X^2^, X^3^* space when attention control is absent - 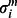 does not depend on .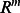. Panel A corresponds to independent modality dynamics (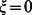); Panel B corresponds to the joint *X^1^, X^2^, X^3^* dynamics with binding stress 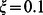. One can see that the ‘bound pattern’ is characterized by a higher level of coherence (see the corresponding power spectrum in Figs. 11A and 11B).

**Figure 10:**
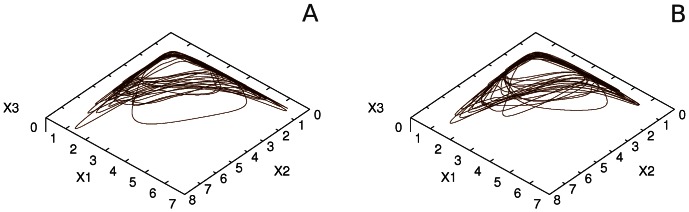
Phase portraits of the joint *X^1^, X^2^, X^3^* steady state dynamics under the top-down attentional control (attentional control dynamics corresponds to the modulation of behavior originated by choosing 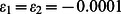). Panel A is an image of unbound modalities (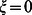), and panel B is an image of “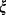-bound modalities” (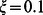). By comparing Figs. 9 and 10 we can say that the attentional control is able to better integrate modalities than 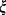-binding (both patterns A and B in this figure have higher coherence than the pattern in Fig. 9B).

**Figure 11:**
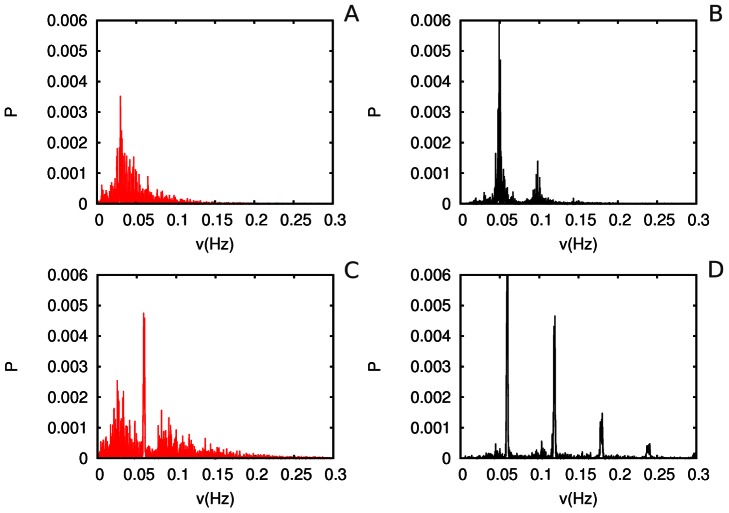
Power spectrum of the activity of 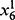 with *t* = 5000 corresponding to uncoupled modalities for A and C and bound with 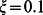 for B and D. Attention dynamics are absent in A and B but they are present in C and D corresponding to the modulation behavior originated by choosing 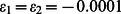. The analyses of these spectra support the result that attention ordered multimodal perception (see Fig. 9 and Fig. 10).

In our formulation, this inhibitory binding is represented by the connection matrix 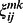 in the equations above. In what follows, we compare (i) the features of the interaction of three sensory modalities with inhibitory binding but without attentional modulation, (ii) interaction between these modalities without inhibitory binding but under attentional modulation, and, finally, (iii) with both inhibitory binding and attentional modulation. It is important to emphasize that the dynamics of individual modalities without inhibitory binding or attentional control are highly irregular. Figure 9 represents the dynamical images without the top-down attentional control in the case of non-interacting modalities (panel A) and bound modalities (panel B). For comparison we show in Fig. 10 the same cases with attentional control. The power spectrum of individual modalities is represented in Fig. 11 (the parameters are indicated in the figure captions).

One can see in these figures that attention orders the modality interactions. This result seems to be very general. In particular, authors in [56] have suggested that attention facilitates the creation and maintenance of novel color-shape bindings in the visual periphery; without attention, binding is less effective.

When the stable limit cycle is disposed in the vicinity of the closed heteroclinic chain (as shown in Fig. 4E and 6), the attentional modulation is very strong. The time that the system spends in the vicinity of the metastable states is very sensitive to the distance in the transverse direction to the heteroclinic chain and even to a very low level of noise (see [20], [57]). Because of this, the time interval between attention switching from one modality to another one in a wide area of the control parameters is a random number that in average becomes longer if the distance in the transverse direction to the heteroclinic chain becomes smaller.

### Strategic decision making

**Figure 12:**
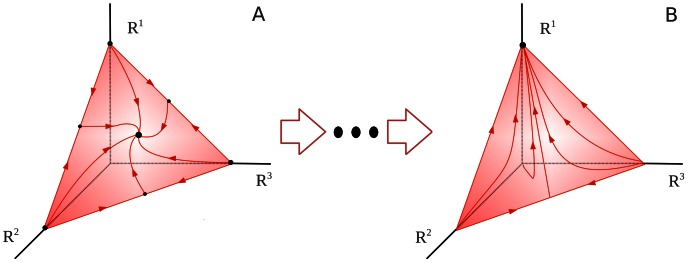
The attention dynamical system (4) in the case of *M* = 3. Panel A is topologically similar to Fig. 4 B – coexisting tasks each of them requiring only a little attention. Panel B corresponds to the case when, independently of the initial condition, only one specific modality attracts all attention resources – ‘winner take all’ attentional regime (for the details of the sequence of bifurcations from A to B, see [51]).

Decision performance and attentional control are two fundamental processes through which we select, respectively, appropriate actions or sources of information. These processes are strongly interconnected. Suppose you are driving a car, half-listening to the local news on the radio simultaneously and receiving a call on your cell phone. Suddenly, you have understood the message on the radio – a car accident just happened a mile ahead of you. A newscaster is describing the situation to the drivers in the vicinity of the accident to help them figure out a way to avoid the cordoned area. Evidently, you have to change the strategy for your behavior: you have to turn off the cell phone, stop the car before you plan a new route and concentrate on the newscaster advice that is repeated from time to time. Here we show how one can describe such strategy modification mathematically in the framework of the dynamical model (4), (6). The multitasking behavior before you got the message is the regime of the divided attention. In the phase space of (4) such regime is represented by the stable fixed point on the simplex (see Fig. 12A). When the newscaster announcement starts, the parameter *C^m^* (for simplicity suppose *m = *1) makes the inhibition of modes *R^2^* and *R^3^* by *R^1^* stronger (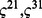 become larger). One can see the corresponding global bifurcation in Fig. 12B – instead of a multimodal fixed point, the system is represented by just one global attractor: a stable node on the axis *R^1^*. Such kind of dynamics is usually known as ‘winner-take all’ (WTA). It corresponds to the focusing of full attention on the performance of just one task – deciding a new driving route based on the newscaster advice and your previous knowledge and experience. The two other modalities *X^2^* and *X^3^* are just suppressed because they have no excitation: 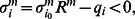 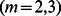.

It is important to emphasize that here we are talking about decision performance that corresponds to a change of strategy for behavior, not just choosing the state like in the multistability case (c.f. Fig. 4A). The later can be described as the jumping through the boundary of the basin of one attractor to the basin of another one. In fact, it is a simple change of the initial condition but the dynamics of the system is still the same. In the case analyzed above, the task and the corresponding performance are absolutely different. The decision making means choosing a functional structure of the interactive modes by changing the set of exciting neuronal clusters and their inhibitory connections (the architecture of the functional networks which depends on the stimulus). Then the overlapping of the attention and decision making networks is well established. For example, parietal neurons encoding saccade motor decisions also carry signals of attention (perceptual selection) that are independent of the metrics, modality and reward of an action. Gottlieb and Balan have proposed that attention implements a specialized form of decision based on the utility of information [58]. Oculomotor control depends on two interacting but distinct processes: attentional decisions that assign value to sources of information and motor decisions that flexibly link the selected information with action [58].

An important aspect of our model is that attention is mediated by changing the dynamics (attractor manifold) of competing multi-modal sensory representations. Effectively, this can be regarded as changing the attractor manifold in a state dependent fashion – in other words, in a way that depends upon the states of higher (attentional) parts of our distributed network. This necessarily induces a separation of temporal scales, in the sense that the fast dynamics at lower levels are governed by slower changes in attentional dynamics – which themselves respond to switches in cognitive sets (modeled by our cognitive variables). The theme of state dependent changes in control parameters fits very comfortably with recent proposals for how attention is mediated during hierarchical Bayesian inference or predictive coding. In these models, the attentional modes (top-down effects) change interactions among neuronal states in the low levels through modulating postsynaptic gain, which encodes the precision of sensory information (see also [42]). The main results of the analysis of the attention model discussed in this paper are summarized in Table 1.

**Table 1:** Summary of the attention model analysis.

Result	Comments
*Dynamical origin of the information processing resource limitation*	*In the state space, the number of metastable states (information items) in whose vicinity robust informational trajectories are located is finite*
*Intrinsic instability of the cross-modality attentional control*	*The inhibitory competition of different attentional modalities is responsible for oscillatory instabilities: the static regime (a stable fixed point) becomes unstable and a limit cycle emerges*
*The time of attention focusing depends on the strength of the sensory input or of the intrinsic informational signal*	*The time during which the system is focused on a given modality explosively increases with the strength of the stimuli corresponding to this modality*
*Attentional dynamics is able to bind multimodality processing*	*Attentional control dynamics can be ordered in time, for example, different sensory modalities perceived simultaneously maintain the order of the information items*
*Strategy changing of attentional control is related to the bifurcation of the attentional dynamics*	*This is the dynamical origin of the decision-making process that, in fact, is a controllable bifurcation of the attention strategy*

## Discussion

Recent experimental results show that transient sequential dynamics underlies many aspects of information processing in the brain. Novel theoretical frameworks are thus needed to represent and characterize phenomena arising within this type of activity in the nervous system. In this paper we have built a nonlinear dynamical theory of multitask attentional control of sequential perception/cognition in multiple interacting brain networks. The presented bifurcation analyses provide us explicit predictions and bridge between neuronal mechanisms (network parameters) and cognitive strategies that finally determine the sequential behavior.

In the last few years, the research on attentional control has produced results using computational models of specific neuronal and cognitive mechanisms. Such modeling has used elements of dynamical theory (see for rev. [59], [60]). Most of the interest has been raised by: (i) analyses of the relationship between attention and plasticity including sequential learning and memory; (ii) attention and timing coordination; (iii) attentional control and personality/aging; and (iv) interaction of attention and emotion, including anxiety and psychiatric disorders. Important questions remain: 1. What are the dynamical mechanisms responsible for the robust perception of transient sequential multimodal information and how sequential WM is related to switching attention?; 2. How does learning functionally reorganize attentional brain networks for temporal multimodality perception?; 3. Do emotion and cognitive attention use common information resources?; 4. What does a psychiatric attentional disorder mean from the dynamical point of view? The dynamical model of attentional control of several simultaneous modalities that we have formulated above is directly applicable for addressing these questions and can be generalized to address other problems in the context of sequential transient cognitive dynamics. Let us discuss here two subjects in more detail.

### Sequential perception-cognition binding, learning and memory

The problem of cross-modal interactions under attentional control has been first raised in the binding of sensory modalities - visual, auditory, tactile, olfactory, gustatory [44], [61]. In the last years the interest of cross-modal cuing between perception and cognition has increased. For example, how sequential information across perception, memory and action planning under attentional control is integrated is now a central question in cognitive neuroscience and language perception [62]–[65].

Consider, as an example, the ‘cocktail-party problem’, i.e., speech recognition in the presence of many sequential sources of useless information [66]. People with only one functional ear, seem much more disturbed by interfering noise than people with two healthy ears. But, even without binaural location information, we can selectively attend to one particular speaker if the pitch of his/her voice or the topic of the speech is sufficiently distinctive and semantically understandable (see [67]). Mesdgarani and Chang found that task performance is well predicted by a rapid increase in attention-modulated neural selectivity across cortical responses [68]. Their findings demonstrate that the cortical representation of speech does not simply reflect the external acoustic environment, but the perception is also based on relevant cognitive modalities like the listener’s performed goal, attention and WM.

Both attention and WM are processes that can be trained. It is known, for example, that musicians have a greater ability to hear speech in noisy environments and to remember sounds [69]. These advantages and specifically the features of auditory WM are a consequence of musical training that can be transferred from the music to the language domain.

WM and attention are characterized by their own sequential dynamics but the networks of the brain that support these two processes are neuro-anatomically overlapping [70]. This overlap can explain the extending of the WM capacity by correlated attention dynamics that optimize common information resources. It can also explain the similarity of inhibitory mechanisms of sequential dynamics underlying the robustness of both attention and memory processes and their plasticity. WM capacity is determined as the number of information items that can be recalled in sequential order without mistakes. This means that the capacity coincides with the length of the item chain like a trajectory in the state space of the WM excitatory/inhibitory network when this chain loses its stability under the action of noise. As it has been shown, this length depends on the topology and strength of the inhibitory connections within the WM networks [4]. The overlapping of the connections in WM and attentional networks can make the effective inhibition stronger and thus the length of the stable WM chain – the capacity – larger.

How does the topology of the inhibitory networks which warranties the sequential switching dynamics appear? A modeling experiment was performed to answer this question [35], [71]. Starting with a model circuit consisting of 100 rate model neurons randomly connected with weak inhibitory synapses, new synaptic strengths were computed using Hebbian learning rules in the presence of weak noise. The neuron activity rates satisfied an equation similar to (4). The matrix of inhibitory connections dynamically changed according to a set of plasticity rules. After the self-organization phase, the network displayed stable sequentially switching dynamics. Such sequential competitive dynamics can also be the result of local self-organization in networks of spiking model neurons that exhibit spike-timing dependent plasticity [72] with inhibitory synaptic connections. These mechanisms of self-organization can be appropriate for networks that generate not only rhythmic sequential activity but also robust transient sequences [73]. This can be important for modeling temporally changing WM sequences [74]. Recent experiments provide evidence that learning and plasticity may have common mechanisms on all information processing levels from perception to decision making (see [75]).

### Interval timing control and sequential attention switching

Because attention switching depends on the environmental inputs and/or intrinsic signals related to cognitive task planning, we can ask: who is first, WM or attention, to generate time intervals between sequential switching events? There is no unique answer to this question yet [76].

Let us discuss a specific cognitive task - music performance. Based on many experimental results one can hypothesize that WM is first used to send the signal to attentional networks and modulate time intervals to produce correct rhythms. WM is a dynamical multiscale process that is supported by networks in prefrontal, parietal and subcortical brain regions including the striatum. It is known that frontostriatal circuitry is involved in the ability to process temporal intervals [77] and overlaps with the attentional corticostriatal timing network [74], [78], [79]. In [80] the authors have proposed that, along with the prefrontal cortex, the striatum plays an important role in cognitive control of memory retrieval. In particular: (1) the striatum modulates the re-encoding of retrieved information items according with their expected utility (adaptive encoding), (2) the striatum selectively admits information into WM that is expected to increase the likelihood of successful retrieval (adaptive gating), and (3) the striatum enacts adjustments in cognitive control based on the outcome of retrieval (reinforcement learning). Based on this knowledge we can focus on the interval timing generation in striatum which participates in WM dynamics and thus transform cognitive information about interval timing to attentional control networks.

The striatum is composed of spiny neurons with inhibitory collaterals forming a sparse random asymmetric network and receiving excitation from the cortex [81]. Ponzi and Wickens showed by simulating the striatal inhibitory network that cells form assemblies firing in the form of sequential coherent episodes and generate temporal patterns with characteristic timescales even if the external excitatory forcing is constant [37]. These results support a new view on sequential information processing in the brain [18]. In this regard, the striatum neuronal inhibitory motif can be analyzed as a modeling circuit to test the hypothesis about the dynamical origin of timing control in such sequential processing.

It is important to emphasize that the dynamical approach that we suggested above (see Table 1) can also be useful for understanding and predicting attentional control processes in the context of mental disorders (for a discussion about the connection of emotion and neurobiology trough dynamical system theory see [82]). For example, it is well known that obsessive-compulsive disorder (OCD) appears to be associated with an attentional bias favoring threatening information, as well as reduced levels of attentional inhibition [83]. Keeping in mind that OCD is a process with randomly switching attention between performing a cognitive task and an emotional ritual [84], one can generalize model (4), (6) for the description of attentional control dynamics in the presence of OCD by taking into account one more modality related to the ritual processing. Preliminary analyses show that OCD destroys the process of transformation of attentional control from one type of dynamics to another one – i.e. destroys the ‘healthy bifurcations sequence’.
